# Response to “Comment on: Machine Learning for Understanding and Predicting Injuries in Football”

**DOI:** 10.1186/s40798-024-00751-3

**Published:** 2024-07-29

**Authors:** Aritra Majumdar, Rashid Bakirov, Tim Rees

**Affiliations:** 1 Department of Rehabilitation and Sport Science, Faculty of Health and Social Sciences, Bournemouth University, Dorset House, Talbot Campus, Fern Barrow, Poole, BH12 5BB UK; 2 Department of Computing and Informatics, Faculty of Science and Technology, Bournemouth University, Dorset House, Talbot Campus, Fern Barrow, Poole, BH12 5BB UK

Dear Editor,

We acknowledge the Letter to the Editor by Bullock and colleagues [1] regarding our article “Machine learning for understanding and predicting injuries in football” [2], and appreciate the opportunity to respond. In our Leading Article [2], we outlined the topics of sport injury and machine learning, before describing examples from the literature that had used machine learning to examine the workload-injury relationship in football. Our aim was to “aid readers both from sport science and machine learning communities in their understanding of sports injury articles employing machine learning” (p. 2) [2]. We concluded: “the myriad ways machine learning can be employed can also lead to difficulty in synthesising the current research evidence into an overall, unified, conclusion. Indeed, there remain questions as to the utility of these models for real-world application” (p. 8) [2].

Given the above, we were confused as to the content and purpose of the letter [1]. The letter either (a) raised points with which we have not disagreed, backed by citations to the letter authors’ own work, (b) made general observations about machine learning, or (c) countered points we never made—strawman logical fallacies.

The letter made five key points [1]. The first point—that we claimed the models that we reviewed in our Leading Article [2] were “quite sound”—is untrue. The letter authors [1] noted that the machine learning studies we reviewed were rated by them in a previous article [3] as having a “high or unclear risk of bias”. We made it quite clear [2] that the machine learning *techniques* employed were legitimate, but that “there is considerable variability in study design and analysis” (p. 7), and “greater detail regarding the machine learning approaches employed would help any objective assessment of their contribution towards better understanding the workload–injury relationship” (p. 7). Thus, through our Leading Article [1]—not a systematic review—we arrived at roughly the same conclusion. Indeed, we wrote at length of our criticisms of the studies we reviewed, which included a lack of detail regarding the machine learning processes followed, such that they “limit a systematic evaluation of findings and the drawing of a unified conclusion” (p. 1) [2].

The second point—that we claimed that machine learning studies demonstrate causality—is also untrue. Nowhere in the article did we discuss causality or try to unpick cause-effect. We consistently used the term workload-injury *relationship*. One wonders, then, why the letter authors felt compelled to deliver such a clichéd, stats-class admonition; for further discussions on this topic, see [4–6].

The third point—that we promoted data balancing and classification models—is similarly untrue. Rather than “promoting”, we noted that data balancing is used in machine learning, describing in the highlighted papers where data balancing had been employed [2]. We would agree that data balancing can have unintended consequences. Over-detection of medical conditions and subsequent over-treatment is one obvious example. The above notwithstanding, data balancing methods may still be critical in such contexts; for example, two recent articles demonstrated that data balancing improved the performance of machine learning models in diagnosing patients with metabolic dysfunction-associated steatohepatitis [7] and cerebrovascular diseases [8]. We also noted (rather than promoted) that the papers highlighted in our leading article had used classification models. Despite the letter’s criticisms, many classification models also provide options to examine probability and risk scores [9].

The fourth point—that we advocated the use of some evaluation metrics at the expense of others—is also untrue. We did not state that certain metrics “should be used” [2]. We noted that many evaluation metrics are used, highlighting those metrics in the papers we reviewed, explaining their meaning, purpose, and limitations. It is true that we did not explicitly highlight “calibration”—something not mentioned in the papers we reviewed. More to the point, part of our Leading Article’s [2] purpose was to “highlight (and to an extent de-mystify) the machine learning process” (p. 8) for non-experts. It was not designed as a treatise on machine learning. The argument that we did not mention calibration could be extended to wondering why we did not mention a host of other potentially important aspects of evaluation [10], such as the confusion matrix, Matthews correlation coefficient, and threat score.

We agree with the letter’s fifth point—that the papers we reviewed in our Leading Article did not use external validation [2]. It is true that without “external validation for such models it is impossible to know whether or not they will be useful to practitioners with different clubs”. It is a moot point, however, whether external validation is always necessary, and whether a universally applicable model for all clubs is desirable or even a logical endpoint of such research. In fact, it is precisely because different clubs use different training regimes, under different coaches, with different athletes, that a universal model would likely prove to be of little practical utility across all clubs. For example, a club may choose to play a distinctive, high tempo pressing style of football or a more strategic, defensive approach, potentially coupled with higher-than-average training volumes. To accurately predict injuries in such specific settings may mean developing predictive models that would not translate well to clubs with different styles of playing and training. Relatedly, we recently reproduced with our own data [11] the analysis strategies from two of the papers [12, 13] highlighted in our Leading Article, observing sizeable discrepancies in the best models.

Finally, the letter noted, in passing, other issues that were not further expanded upon [1]. We shall thus not address these here, but again refer readers to our point that our article was not a treatise on machine learning.

Given the above, as a target for criticism, then, the choice of our Leading Article [2] seems strange. Although we welcome constructive peer review and the opportunity to provide clarifications of our work, we stress the importance of paying attention to the specifics of the article content, and making factual and unbiased observations of others’ work.
